# The Influence of Negative Affect on Working Memory Updating of Food Stimuli Among Adolescents With Anorexia Nervosa

**DOI:** 10.1002/eat.24367

**Published:** 2025-01-03

**Authors:** Meital Gil, Yael Latzer, Noa Tziperman, Dan Farbstein, Helene Sher, Noam Weinbach

**Affiliations:** ^1^ School of Psychological Sciences University of Haifa Haifa Israel; ^2^ Faculty of Social Welfare and Health Sciences University of Haifa Haifa Israel; ^3^ Eating Disorders Institution, Psychiatric Division Rambam, Health Care Campus Haifa Israel; ^4^ Pediatric Psychosomatic Department, Safra Children's Hospital Sheba Medical Center Tel Hashomer Israel; ^5^ Department of Psychiatry Soroka Medical Center Beer Sheva Israel

**Keywords:** adolescents, anorexia nervosa, food, *N*‐back, negative affect, working memory updating

## Abstract

**Objective:**

Difficulty updating information in working memory has been proposed to underlie ruminative thinking in individuals with anorexia nervosa (AN). However, evidence regarding updating difficulties in AN remains inconclusive, particularly among adolescents. It has been proposed that exposure to negative emotion and disorder‐salient stimuli may uniquely influence updating in AN. This study examined the influence of exposure to negative emotion on updating of food and non‐food stimuli among adolescents with AN.

**Method:**

The study included 41 female adolescents with restrictive AN (*M*_age_ = 15.2) and 43 female controls (*M*_age_ = 16.9) who performed a modified version of the *N*‐back task requiring updating of food and non‐food content after supraliminal exposure to emotionally neutral and negative images.

**Results:**

Medium‐to‐large effects revealed significantly higher updating errors among adolescents with AN compared to controls for food targets and after exposure to negative stimuli. In contrast, during exposure to both negative images and food targets, the groups performed similarly, as this condition also increased updating errors among controls. Additionally, a higher proportion of updating errors was associated with greater eating disorder symptoms severity.

**Discussion:**

The findings indicate that exposure to negative emotion and food stimuli separately compromise working memory updating in adolescents with AN. These results underscore the role of environmental triggering cues in compromising working memory updating in AN and highlight the importance of considering ecological cues when evaluating cognitive functioning in eating disorders.

1

**Summary**

- Poor working memory updating may underlie ruminative thinking in anorexia nervosa (AN), but findings have been inconsistent.
- This study found impaired updating in adolescents with AN during exposure to negative affect and food content.
- The results highlight the role of triggering environmental cues in modulating cognitive abilities in AN.

## 2 Introduction

Working memory updating (referred to as ‘updating’ henceforth) is the cognitive ability that allows replacing old, no longer relevant information in working memory with newer, more relevant information (Morris and Jones 1990). Updating of working memory is a dynamic process and involves monitoring information in response to changing enviromental demands and task requirements (Friedman and Miyake 2017). Given its broad implication for adaptability to environmental demands, difficulties in updating have been proposed to contribute to and maintain mental health problems (Fosco et al. 2020; Friedman et al. 2018; Gustavson and Miyake 2016; Nikolin et al. 2021; Snyder, Miyake, and Hankin 2015; Yuan et al. 2024).

In eating disorders, inefficient updating is theorized to underlie difficulties filtering out disorder‐relevant information from working memory and thus results in ruminative, intrusive, and repetitive thinking about weight and shape (Forester et al. 2023). To date, research on working memory in eating disorders has yielded mostly inconsistent findings (for reviews see Brooks et al. 2017; Forester et al. 2023). Although there is some support for a deficit in phonological working memory (i.e., the storage of verbal and auditory information) in individuals with eating disorders, and particularly anorexia nervosa (AN), evidence regarding abnormalities in visuospatial working memory (i.e., the storage of visual and spatial information) remains inconsistent (Dahlén et al. 2022).

For instance, several studies that assessed updating of visuospatial information in AN reported poorer updating abilities in adults with AN compared with controls (e.g., Adamski et al. 2017; Dickson et al. 2008), some showed superior updating in adults with AN (e.g., Brooks et al. 2012; Israel et al. 2015), and others did not find a difference in updating abilities between adults or adolescents with AN compared to healthy controls (Castro‐Fornieles et al. 2010; Lao‐Kaim et al. 2014). It has been proposed that a potential reason for these inconsistencies is that most studies did not consider situational factors that could influence updating abilities. For example, exposure to emotionally triggering stimuli, such as negative emotional images or disorder‐salient stimuli (e.g., high‐calorie foods) that may uniquely affect performance. Instead, most studies have relied on traditional working memory updating tasks using neutral stimuli, such as letters, shapes, or numbers (Forester et al. 2023).

Over the past decades, multiple studies in various domains stressed the importance of triggering environmental cues and other situational factors in modulating cognitive abilities in psychopathologies (Günther et al. 2021; Suslow et al. 2020). In the field of eating disorders, studies report that exposure to disorder‐salient cues such as food or body content modulates various cognitive functions (Forester et al. 2023; Lloyd and Steinglass 2018; Ralph‐Nearman et al. 2019; Weinbach, Lock, and Bohon 2019). To date, only a few studies have assessed how disorder‐salient content influences updating abilities in AN. One study reported that exposure to subliminal food images compromises updating in adults with AN (Brooks et al. 2012). However, a different study that also included exposure to food stimuli within an updating task, did not find a unique influence of such stimuli on updating performance among adults with AN (Dickson et al. 2008).

Another situational factor that could have a substantial impact on cognitive abilities is affective state. Negative affect is an important variable in the development of and maintenance of AN (Engel et al. 2016; Stice et al. 2021; Stice, Desjardins, and Rohde 2022). It has been shown that momentary increases in negative affect precede restrictive eating episodes in AN (Engel et al. 2016). In one study that examined how aversive images influence updating abilities in adults with AN, there was no difference in updating abilities between AN and control groups when aversive or neutral images were cued before participants engaged in updating (Brooks et al. 2012). Nevertheless, other studies have reported that exposure to aversive images compromises updating among anxious individuals and those with depression (Bruning, Mallya, and Lewis‐Peacock 2023; Ladouceur et al. 2009). To conclude, there are limited data on the potential influence of negative emotion on updating abilities in AN and no data on the impact of negative emotion on updating of food content in AN. Assessing how different environmental cues interact to influence cognitive abilities in AN could provide a more ecological understanding of how impaired cognitive functioning acts to maintain symptoms of AN.

An additional gap in understanding updating abilities in individuals with AN is that most studies were conducted using samples of adults, leaving adolescents with AN relatively understudied. It is important to assess cognitive functioning in this age group because younger patients are characterized by shorter illness duration, potentially making them less prone to cognitive deficits associated with prolonged illness (Lao‐Kaim et al. 2014). Although several studies evaluated working memory performance among adolescents with AN (Kjærsdam Telléus et al. 2016; van Noort et al. 2016), only one study assessed the updating function of working memory and found no difference in performance between adolescents with AN and controls (Castro‐Fornieles et al. 2010). However, this study did not incorporate disorder‐salient stimuli nor manipulated exposure to other emotional stimuli.

The goal of the current study was to assess the influence of negative affect on the ability to update food and non‐food content in working memory in adolescents with AN compared to adolescents without an eating disorder. The sample included adolescents who received a diagnosis of AN‐restrictive type, but were not severely malnourished while participating in the study in order to mitigate potential effects of malnourishment on cognitive functioning (Hemmingsen et al. 2021). All participants underwent a well‐established working memory updating task (i.e., *N*‐back task) that we modified to include exposure to negative or neutral emotional stimuli while being requested to update food and non‐food images. We hypothesized that adolescents with AN would show poorer ability than controls in updating food compared to non‐food content and that this effect would further escalate when negative emotional stimuli were embedded within the task.

## 3 Method

### 3.1 Participants

The study recruited 42 adolescents with restrictive AN and 47 controls (see power analysis below). Three participants were excluded from the control group for failing to produce a response on more than 50% of the trials. Moreover, data from one participant with AN and one participant from the control group were excluded from the analysis due to high error rates in the N‐back task (above 3 standard deviations from their group mean). Accordingly, the final sample included 41 adolescents with AN and 43 controls. All participants were females (see clinical and demographic data in Table 1). All adolescents with AN were receiving inpatient care for AN at different levels of treatment. Most of the participants (88%) were partially weight restored while participating in the study (i.e., Expected Body Weight > 85%). However, all individuals were diagnosed with full AN at admission. A power analysis using G*Power (Faul et al. 2007) showed that for a power of > 80% with an a priori alpha set at 0.05 and an expected medium sized effect of *η* ^2^_p_ = 0.06, 39 participants are required in each group for detecting within‐between subject variables interactions.

**TABLE 1:** Clinical and demographic measures of the participants.

	AN (*n* = 41)	Controls (*n* = 43)	*p*‐value	Cohen's *d*
Age (years)	15.2 (1.6)	16.9 (1.4)	< 0.001	−1.15
Illness duration (months)	23.15 (10.46)	—	—	—
A report of previous hospitalizations (%)	31.7%	—	—	—
Use of psychotropic medications (%)	75.6%	0%	—	—
Lowest BMI	16.53 (1.65)	—	—	—
%EBW	95.41 (10.08)	103.55 (16.42)	0.01	−0.58
Wechsler IQ scaled score—Vocabulary	10.79 (3.05)	12.11 (2.96)	0.05	−0.44
Wechsler IQ scaled score—Matrices	10.08 (2.56)	10.95 (3.24)	0.18	−0.3
EDE‐Q‐Global score	4.28 (1.09)	1.32 (1.28)	< 0.001	2.49
DASS‐depression	12.78 (4.81)	3.95 (4.29)	< 0.001	1.94
DASS‐anxiety	10.78 (5.44)	2.28 (2.83)	< 0.001	1.97
DASS‐stress	13.46 (4.28)	4.39 (4.39)	< 0.001	2.09
RRS‐global score	63.27 (12.44)	37.14 (12.25)	< 0.001	2.12
Non‐ED diagnoses (%)				
Major depressive disorder/dysthymia	34.15%	4.65%		
Anxiety disorder	36.58%	2.32%		
Obsessive‐compulsive disorder	14.63%	2.32%		
Post‐traumatic stress disorder	7.32%	0%		
Attention deficit hyperactivity disorder	2.44%	2.32%		

*Note*: Standard deviations appear in parenthesis.

Abbreviations: BMI = body mass index; DASS = Depression Anxiety Stress Scales (higher scores indicate greater severity); %EBW = % expected body weight; EDE‐Q = Eating Disorder Examination Questionnaire (higher scores mean greater symptoms severity); RRS = Ruminative Response Scale (higher scores represent greater tendency to ruminate).

### 3.2 Recruitment

Adolescents with AN were recruited from two eating disorders inpatient centers in Israel (“Soroka” and “Rambam” medical centers) between the years 2020 and 2023. Adolescents in the control group were recruited via advertisements on social networks. Inclusion criteria for the patient group were (a) age within the range of 12–18 (b) a diagnosis of restrictive AN based on the Diagnostic and Statistical Manual of Mental Disorders, fifth edition (American Psychiatric Association 2013) and (c) BMI > 12. Inclusion criteria for the control group were: (a) age within the range of 12–18, (b) absence of an eating disorder currently or in the past. We did not exclude controls for having any other psychopathology to obtain a sample that better represents a community sample of adolescents. Exclusion criteria for both groups were the presence of any neurological illness, brain injury, or trauma that could interfere with neurocognitive functioning, as assessed via self‐report.

### 3.3 Clinical Assessment

All participants underwent the MINI‐KID International Neuropsychiatric Interview (Sheehan et al. 2010) for assessing the presence/absence of an eating disorder as well as other psychopathologies including major depression, dysthymia, social anxiety disorder, obsessive compulsive disorder, post‐traumatic stress disorder, attention deficit hyperactive disorder, and generalized anxiety disorder. In the eating disorder clinics, the MINI‐KID was administered by a clinical psychologist or psychiatrist. In the control group, a trained research coordinator, supervised by a licensed clinical psychologist (NW), conducted the interviews.

### 3.4 Measures

*Expected Body Weight* (%EBW): Height and weight of adolescents with AN were measured on the day of the assessment using a weight scale and a stadiometer. Height and weight of adolescents without AN was based on self‐report. Expected body weight (%EBW) was then calculated based on the 50th percentile for height, age, and gender from the Centers for Disease Control and Prevention (CDC) charts.

*Intelligence assessment*: Potential differences in IQ between the groups were assessed using the vocabulary and matrices subsets of the Wechsler Adult Intelligence Scale (WAIS; for participants aged 16 or above; Wechsler 1997) and Wechsler Intelligence Scale for Children (WISC; for participants under the age of 16; Wechsler 2004), with raw scores transformed to scaled scores based on participants' age.

*Self‐report questionnaires*: Eating disorders symptoms severity was assessed using the Eating Disorder Examination Questionnaire (EDE‐Q; Fairburn and Beglin 1994; Cronbach's alpha in our study was 0.97). Symptoms of depression, anxiety, and stress were assessed using the Depression Anxiety Stress Scales (DASS; Lovibond and Lovibond 1995; Cronbach's alpha for the depression, anxiety and stress scales in our study was 0.92, 0.92, and 0.91, respectively), and self‐reported rumination tendency was assessed using the Ruminative Response Scale (RRS; Treynor, Gonzalez, and Nolen‐Hoeksema 2003; Cronbach's alpha in our study was 0.95). These questionnaires were used to assess their potential correlations with the primary outcomes.

### 3.5 Emotion/Food N‐Back

**FIGURE 1:**
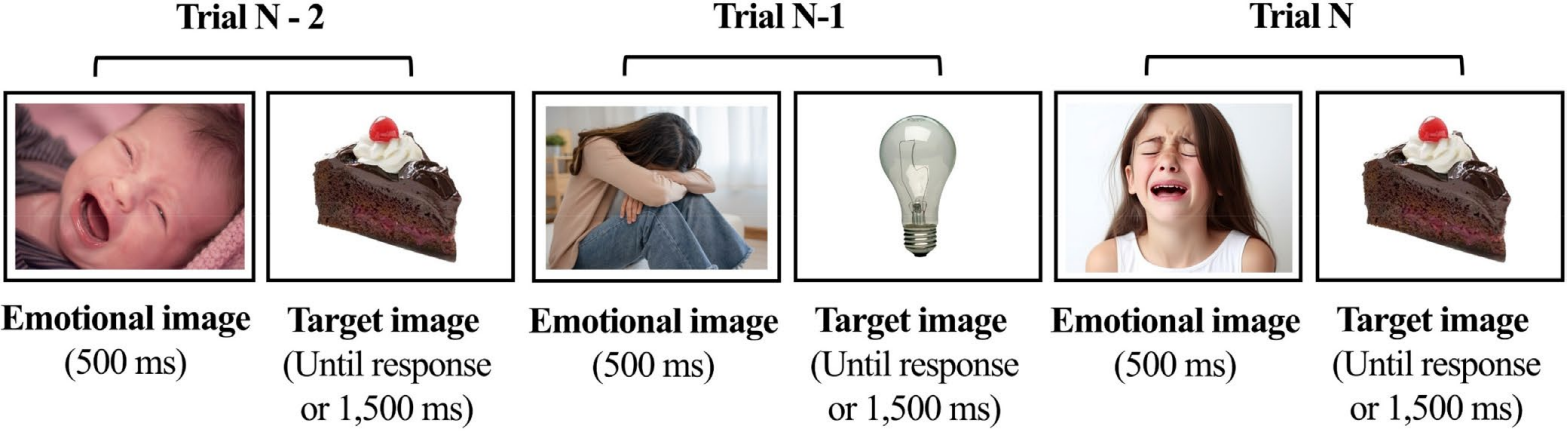
An example of three consecutive trials in a negative emotion block. In the 1‐back condition, participants had to press the ‘z’ key on trial N (i.e., cake) because the target image in trial N‐1 (i.e., light bulb) was not identical to that in trial N. In the 2‐back condition, participants had to press the ‘m’ key on trial N because the cake image is identical to the cake that appeared in trial N‐2. This example is taken from the negative emotion block condition because all emotional images between the target stimuli are negatively valenced.

In the task, participants were presented with a continuous sequence of high‐calorie food and non‐food images. For each stimulus, participants were asked to decide if the stimulus was identical to that presented earlier. In a 1‐back condition participants were instructed to press the ‘m’ key on the keyboard if the currently presented stimulus was identical to the one that appeared one trial earlier. If it was different, participants pressed the ‘z’ key (key choices were counterbalanced across participants). The 1‐back condition serves as a control condition because it requires only maintaining information in working memory and not updating (Ragland et al. 2002). In a 2‐back condition, participants were required to decide if the currently presented stimulus was identical to the one that appeared two trials earlier. For example, if participants saw a sequence of a cake—light bulb—cake (see Figure 1), they had to press ‘m’ when seeing the second cake. The 2‐back condition requires both maintaining and manipulating information in working memory (i.e., continuously maintaining, discarding, and updating the stimuli held in working memory). The inclusion of food and non‐food images within the task allowed us to separate performance measures for food and non‐food stimuli. The task included four blocks of the 1‐back condition that preceded four blocks of the 2‐back condition. Short practice trials that included a feedback in case of erroneous responses were provided before the 1‐back and 2‐back blocks. Each practice phase included 15 trials. Furthermore, between each food and non‐food target in the task, we embedded neutral or negative emotional pictures. The task included four neutral and four negative emotion blocks in which an emotionally neutral or negative image appeared between the target stimuli (see Figure 1). Overall, the task included two negative and two neutral emotion blocks in each n‐back condition (i.e., 1‐back and 2‐back). The order of the negative and neutral emotional blocks in each *n*‐back condition was counterbalanced across participants. Overall, the task included 480 trials over eight blocks (60 trials in each block). Participants were offered a short break after each block. The sequence of the target stimuli in each block was pseudo‐randomized so that each block included 16 trials that required a hit ‘m’ response and 44 that required a correct rejection ‘z’ response.

*Emotion pictures*: 64 negative and 64 neutral images were selected from the International Affective Picture System (IAPS; Lang, Bradley, and Cuthbert 2008). Neutral images were chosen for their medium valence and low arousal ratings based on normative data (*M*_valence_ = 5.53, SD = 1.38; *M*_arousal_ = 3.61, SD = 1.96). Negative images were chosen based on their low valence (i.e., indicating greater negativity ratings) and high arousal (*M*_valence_ = 3.1, SD = 1.63; *M*_arousal_ = 5.32, SD = 2.14). The study only included negative images that are appropriate for adolescents to view. The neutral and negative images had an equal proportion of human, animal, and object pictures.

*Food and Non‐food pictures*: 64 high‐calorie food images and 64 non‐food images (i.e., household items only) were selected from a food picture database (Blechert et al. 2014). Food images had an equal proportion of sweet and salty foods.

### 3.6 Procedure

The study was part of a larger project that evaluated executive functions in adolescents with AN. Ethical approvals were obtained from the University of Haifa IRB committee (479/20) and the Helsinki Committee at each recruitment site (“Soroka”: 0366‐20‐SOR, and “Rambam”: 0837‐20‐RMB). Once eligible participants were identified, a research coordinator contacted the potential participant and her parents to obtain informed consent. Following the consent process, the adolescent received an online link to the self‐report questionnaires. Then, a research coordinator scheduled a meeting with the adolescent, either at her home (some meetings were conducted via video conference software due to COVID‐19) or at the local eating disorder unit. During this meeting, participants completed an IQ assessment, the MINI‐KID (some participants at the eating disorder units underwent the MINI‐KID either before or after this session), and another executive function task that is beyond the scope of the current study. In a second session, scheduled 7–14 days later, participants completed the Emotion/Food N‐Back task which took between 25 and 30 minutes to complete.

### 3.7 Statistical Analysis

Independent *t*‐tests were used to assess potential differences between the groups on demographic and clinical measures. Response times (RTs) and error rates (including both false alarms and misses) were examined separately in the 1‐back and 2‐back conditions using mixed‐model analyses of variance (ANOVAs) with group (AN/control) as a between‐subject independent variable, and target type (food/non‐food), and emotion block (negative/neutral) as within‐subject independent variables. In line with recommendations regarding analysis of the N‐back task (Meule 2017), we also conducted a separate analysis that separated errors that were due to false alarms (i.e., identifying a target as present when it is not) and miss errors (i.e., failing to detect a present target). Trials in which participants failed to produce a response (6.67%) were removed, and the RT analysis was conducted only on correct response trials (11.23%). Finally, exploratory correlational analyses assessed associations between error rates and clinical measures including eating disorder severity (EDE‐Q global score), self‐reported rumination tendencies (RRS global score), illness duration, and %EBW.

## 4 Results

### 4.1 Clinical and Demographic Measures

Clinical and demographic variables are presented in Table 1. Adolescents with AN had lower %EBW, higher EDE‐Q global scores, higher scores of depression, anxiety, and stress (DASS), higher rumination scores (RRS), and were younger in age compared to controls.

### 4.2 N‐Back Error Rates Analyses

1*‐back*: There were no main effects for target type, *F*(1,82) = 0.00, *p* = 0.99, *η* ^2^_p_ = 0.00, emotion block, *F*(1,82) = 1.16, *p* = 0.28, *η* ^2^_p_ = 0.01, or group, *F*(1,82) = 0.69, *p* = 0.41, *η* ^2^_p_ = 0.00. The three‐way interaction between target type, emotion block, and group was not significant either, *F*(1,82) = 0.29, *p* = 0.59, *η* ^2^_p_ = 0.00.

**FIGURE 2:**
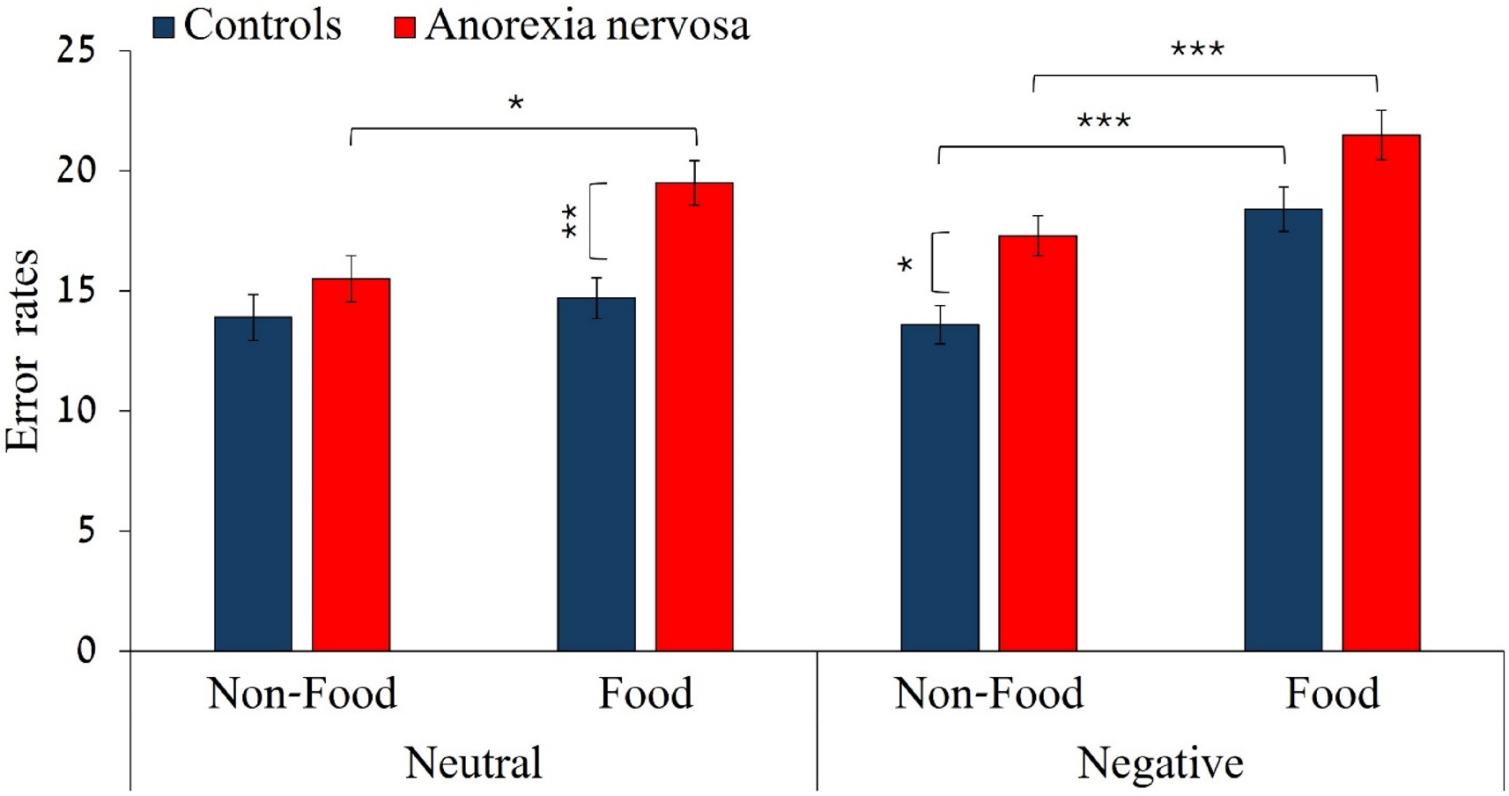
The *y*‐axis represents error rates. The left side of the graph shows results for neutral emotion block and the right side results for the negative emotion block. The *x*‐axis represents the target type (food or non‐food). Blue bars represent results of controls and red bars represent results of adolescents with anorexia nervosa. *p < 0.05; **p < 0.01; ***p < 0.001.

2*‐back*: The ANOVA revealed a significant main effect for target type *F*(1,82) = 38.12, *p <* 0.001, *η* ^2^_p_ = 0.32, demonstrating more errors when responding to food compared to non‐food images. The main effect for emotion block was also significant, *F*(1,82) = 8.76, *p* = 0.004, *η* ^2^_p_ = 0.09, showing more errors on negative compared to neutral emotion blocks. The main effect for group was also significant, *F*(1,82) = 4.87, *p* = 0.03, *η* ^2^_p_ = 0.06, demonstrating a higher error rate among adolescents with AN compared to controls. Lastly, the three‐way interaction between target type, emotion block, and group was also significant, *F*(1,82) = 4.05, *p* = 0.04, *η* ^2^_p_ = 0.05. In support of the hypothesis, planned comparisons (see Figure 2) revealed that after exposure to neutral emotional stimuli, adolescents with AN did not differ from controls in error rates when non‐food targets appeared, *F*(1,82) = 0.74, *p* = 0.39, *η* ^2^_p_ = 0.00, but did make more updating errors than controls when a food target was present, *F*(1,82) = 9.36, *p* = 0.003, *η* ^2^_p_ = 0.10. This was due to an increase in errors among those with AN between the non‐food and food condition *F*(1,82) = 12.6, *p* = 0.001, *η* ^2^_p_ = 0.13, which did not occur for controls *F*(1,82) = 0.59, *p* = 0.45, *η* ^2^_p_ = 0.00. In the negative emotion block, adolescents with AN demonstrated more updating errors than controls for non‐food targets *F*(1,82) = 4.51, *p* = 0.037, *η* ^2^_p_ = 0.05, as expected. However, this difference did not reach significance when food targets followed negative emotional stimuli *F*(1,82) = 2.83, *p* = 0.09, *η* ^2^_p_ = 0.03. Although, as expected, error rates significantly increased between the non‐food and food target condition after exposure to negative stimuli in the AN group, *F*(1,82) = 17.52, *p* < 0.001, *η* ^2^_p_ = 0.18, controls also showed an unexpected increase in error rates when exposed to both negative images and food targets, *F*(1,82) = 26.77, *p* < 0.001, *η* ^2^_p_ = 0.25.

2*‐back*: *misses* vs. *false alarm errors*: Given the overall higher error rates in the AN compared to the control group in the 2‐back condition, we carried out an additional analysis to assess if this group difference results mostly from miss errors (i.e., falsely identifying a target as a non‐target) or false alarms (i.e., falsely identifying a non‐target as a target). Findings show that adolescents with AN had a numerically higher proportion of miss errors (27.17%) compared to controls (24.27%), but this difference did not reach statistical significance level, *F*(1,82) = 0.75, *p* = 0.39, *η* ^2^_p_ = 0.00. In contrast, adolescents with AN had a significantly larger proportion of false alarm responses (14.65%) compared to the control group (9.7%), *F*(1,82) = 6.63, *p* = 0.01, *η* ^2^_p_ = 0.07.

### 4.3 N‐Back RTs Analyses

*1‐back*: The ANOVA revealed a significant main effect for target type, *F*(1,82) = 19.15, *p* < 0.001, *η* ^2^_p_ = 0.19, demonstrating faster RTs when responding to non‐food targets compared to food targets (see Table 2). The main effects for emotion block and group were not significant, *F*(1,82) = 0.06, *p* = 0.80 *η* ^2^_p_ = 0.00, and *F*(1,82) = 0.12, *p* = 0.73, *η* ^2^_p_ = 0.00, respectively. The three‐way interaction between target type, emotion block, and group was not significant either, *F*(1,82) = 3.81, *p* = 0.054, *η* ^2^_p_ = 0.04.

**TABLE 2:** Response times (in millisecond) as a function of emotion block, target type and group.

Trial type (Ype)	Negative emotion block			Neutral emotion block		
	AN (*n* = 41)	Control (*n* = 43)	AN vs. control *p*‐value	AN (*n* = 41)	Control (*n* = 43)	AN vs. control *p*‐value
1‐back—food target	755 (16)	745 (18)	0.66	754 (15)	745 (18)	0.70
1‐back—non‐food target	730 (14)	736 (18)	0.80	746 (15)	728 (18)	0.46
2‐back—food target	805 (17)	798 (16)	0.79	812 (19)	797 (16)	0.52
2‐back—non‐food target	797 (19)	794 (18)	0.89	793 (20)	768 (16)	0.32

*Note*: Standard errors are presented in parenthesis.

Abbreviation: AN, anorexia nervosa.

*2‐back*: The ANOVA revealed a significant main effect for target type, *F*(1,82) = 10.23, *p* < 0.01, *η* ^2^_p_ = 0.11, replicating the results of the 1‐back analysis, namely, slower RTs for food compared to non‐food targets. The main effects for emotion block and group were not significant, *F*(1,82) = 1.22, *p* = 0.27, *η* ^2^_p_ = 0.01, and *F*(1,82) = 0.28, *p* = 0.59, *η* ^2^_p_ = 0.00, respectively. The three‐way interaction between target type, emotion block, and group was not significant either, *F*(1,82) = 0.73, *p* = 0.39, *η* ^2^_p_ = 0.00.

### 4.4 Correlations Between Clinical Variables and Error Rates in the N‐Back Task

We assessed correlations between error rates in the 1‐back and 2‐back conditions (i.e., averaged for emotion block type and target type) and eating disorders symptoms severity (EDE‐Q global score), self‐report rumination tendencies (RRS global score), and other clinical variables (illness duration, %EBW). The analyses aggregated both groups to reflect a wide spectrum of eating disorder symptoms severity (see Table 3). The analysis revealed that higher error rates on the 1‐back condition were associated with higher error rates on the 2‐back condition. Moreover, higher error rates on the 2‐back condition were positively correlated with eating disorder symptoms severity. Furthermore, self‐reported rumination tendencies measure and eating disorder symptoms severity were positively correlated. No other correlations reached a significance level.

**TABLE 3:** Correlations between clinical measures and total error rates in 1‐back and 2‐back conditions.

	Total error rates in 1‐back	Total error rates in 2‐back	EDE‐Q global	RRS global	Illness duration^a^	%EBW
Total error rates in 1‐back	—					
Total error rates in 2‐back	0.53***	—				
EDE‐Q global	0.15	0.23*	—			
RRS global	0.08	0.16	0.79***	—		
Illness duration^a^	−0.29	−0.26	−0.1	0.16	—	
%EBW	0.07	0.09	−0.13	−0.06	0.16	—

*Note*: The analysis included all participants who filled in the questionnaires and completed the Emotion‐Food Task Switching Paradigm (*N* = 88).

Abbreviations: EDE‐Q = Eating Disorder Examination Questionnaire; RRS = Ruminative Response Scale; %EBW = % expected body weight.

^a^ Illness duration was calculated only for adolescents with AN.

* *p* < 0.05.

*** *p* < 0.001.

## 5 Discussion

The study assessed the influence of exposure to emotional stimuli on working memory updating of food and non‐food content among adolescents with restrictive AN. In the 1‐back condition that did not require working memory updating, there was no difference in error rates or RTs between adolescents with AN and controls. In the 2‐back condition that required updating, poorer updating performance among adolescents with AN compared to controls was observed in terms of error rates, but not RTs. More importantly, exposure to either food content or negative emotion independently increased updating errors among adolescents with AN compared to controls. Surprisingly, in the condition involving both food content and negative emotion, there was no difference between the groups because this condition also increased updating errors among controls. Secondary analyses revealed that the poorer updating observed in adolescents with AN was primarily due to an increased tendency toward false alarms (i.e., incorrectly identifying a non‐target as a target) rather than misses (i.e., failing to identify a target). A small‐to‐medium association between errors in the 2‐back condition and eating disorder symptoms severity was also observed.

Previous research on working memory updating in adults with AN has yielded mixed results, particularly in visuospatial tasks (Dahlén et al. 2022). Some studies reported poorer updating abilities in AN compared to controls (e.g., Adamski et al. 2017; Dickson et al. 2008), others suggested superior performance (e.g., Brooks et al. 2012; Israel et al. 2015), or no difference at all (e.g., Lao‐Kaim et al. 2014). Given these inconsistencies, it was proposed that embedding disorder‐salient stimuli within working memory tasks might lead to more robust effects (Forester et al. 2023). This study is the first to examine the influence of exposure to emotional stimuli on working memory updating of food and non‐food content among adolescents with AN.

In line with our hypothesis, updating of food content led to more updating errors in adolescents with AN compared to adolescent controls, regardless of exposure to negative emotion. Specifically, in the neutral emotional block that does not involve exposure to negative stimuli, adolescents with AN did not differ from controls when updating non‐food targets and had more updating errors only for food targets. Since the only difference between these conditions was the presence or absence of food targets, this finding highlights the unique influence of food content on impairing updating in AN. This result also aligns with prior research showing that exposure to food stimuli compromises updating in individuals with AN (Brooks et al. 2012). Furthermore, it supports broader literature indicating that disorder‐salient environmental cues uniquely influence cognitive functioning in eating disorders (Forester et al. 2023; Lloyd and Steinglass 2018; Ralph‐Nearman et al. 2019; Weinbach, Lock, and Bohon 2019).

Similarly, exposure to negative emotional content also impaired updating in adolescents with AN independently of exposure to food targets. Specifically, updating of non‐food targets led to more updating errors in AN than controls only after they followed negative images, but not when they followed neutral images. Given that the only difference between these conditions is the presence or absence of negative emotional content, this finding underscores the detrimental effect of negative emotion on updating in AN. This finding converges with previous reports regarding the important contributing role played by negative affect in eating disorder symptoms (Engel et al. 2016).

Contrary to our predictions, in a condition that involved combined exposure to both negative images and food targets there was no difference between the groups in updating errors. Although this condition resulted in the highest error rates in both groups, the lack of a significant group difference was due to a marked increase in errors among controls in this condition, which was not observed in the food‐only or emotion‐only conditions. It appears that the unique combination of both negative images and food stimuli was sufficiently powerful to impair updating even among controls, possibly due to the widespread preoccupation with eating and weight concerns among female adolescents which is also maintained by negative affect (Rodgers and Melioli 2016). Indeed, in some cases, manipulations of environmentally triggering cues in community samples result in disordered eating patterns that mimic those in clinical samples (see review in Devonport, Nicholls, and Fullerton 2017).

Secondary analyses revealed that poorer performance among those with AN in the 2‐back compared to the 1‐back condition was generally reflected in a greater propensity to perform false alarms, namely, a tendency to identify a non‐target stimulus as a target stimulus rather than miss errors (i.e., failing to identify a target). Tendency toward false alarms may reflect exaggerated sensitivity to performance monitoring in adolescents with AN. Specifically, individuals with AN are often characterized by perfectionism that may lead to heightened concerns with making errors (Pieters et al. 2007). This oversensitivity may compromise performance by tending to mark a non‐target as a target. Hence, future research on updating in AN is encouraged to incorporate measures of perfectionism.

When assessing potential associations between self‐reports and behavioral performance, it was evident that there was no association between updating performance and a tendency toward rumination. This is despite research suggesting that updating difficulties may underlie ruminative thinking, as both reflect difficulty disengaging from no longer relevant information (Bruning, Mallya, and Lewis‐Peacock 2023; Joormann and Gotlib 2008). However, poorer updating was associated with eating disorder symptom severity as measured by the EDE‐Q. Since the EDE‐Q assesses eating, weight, and shape concerns, it may capture disorder‐specific rumination tendencies better than a general rumination scale. This suggests that updating performance in tasks incorporating exposure to negative emotion and food stimuli may show stronger associations with eating disorder‐specific rumination tendencies rather than general rumination tendencies. Future studies should include disorder‐specific rumination measures, such as the Ruminative Response Scale for Eating Disorders (RRS‐ED; Cowdrey and Park 2011), to test this hypothesis directly.

The current study offers potential clinical implications. Cognitive training interventions, such as cognitive remediation therapy (CRT), have been proposed to alleviate eating disorder symptoms by targeting cognitive deficits (Tchanturia, Lloyd, and Lang 2013). However, evidence supporting the efficacy of CRT in improving eating disorder symptoms remains limited (Hagan, Christensen, and Forbush 2020). The findings of this study highlight the potential importance of incorporating environmental triggering cues, such as negative emotion and food stimuli, into cognitive training protocols for AN. This approach could enhance the ecological validity of these interventions and potentially result in better outcomes.

Several limitations should be acknowledged. First, our sample was limited to adolescents with the restrictive subtype of AN, which does not fully represent the broader spectrum of individuals with different subtypes of anorexia. Future studies should include participants from both resctrictive and binge eating/purging subtypes to provide a more comprehensive understanding of updating abilities across the spectrum of AN. Furthermore, our study did not explore the effects of different types of emotions on updating abilities. It could be that different emotions elicit different effects on processing of food vs. non‐food stimuli. As such, future studies should examine if various emotion types uniquely impact cognitive processes in adolescents with AN. Finally, adolescents with AN in our study were younger than controls. However, including age as a covariate in the analyses did not alter the significance of the critical three‐way interaction, suggesting that age differences do not fully account for the observed effects.

To conclude, the present study provides novel evidence regarding how working memory updating functions in adolescents with AN. Our findings demonstrate the role played by triggering environmental cues such as negative emotion and food stimuli on working memory updating in AN. Our work further highlights the importance of assessing cognitive abilities while considering disorder‐related situational factors in order to better understand the role played by cognition‐emotion interactions in the development and maintenance of eating disorders. Future studies are warranted to build on these findings and characterize how updating difficulties translate into clinical symptoms such as ruminative and intrusive thoughts about food, which are common in AN and other eating disorders.

## Author Contributions

**Meital Gil:** conceptualization, data curation, formal analysis, methodology, writing – original draft. **Yael Latzer:** data curation, writing – review and editing. **Noa Tziperman:** data curation, writing – review and editing. **Dan Farbstein:** data curation, writing – review and editing. **Helene Sher:** data curation, writing – review and editing. **Noam Weinbach:** conceptualization, funding acquisition, supervision, writing – review and editing.

## Ethics Statement

Ethical approval was obtained from the University of Haifa IRB committee (479/20) and Helsinki committee at each of the two recruitment sites (“Soroka”: 0366‐20‐SOR, “Rambam”: 0837‐20‐RMB).

## Conflicts of Interest

The authors declare no conflicts of interest.

## Data Availability

All data have been publicly available at the Open Science Framework (OSF) and can be accessed at https://osf.io/ms8wt/?view_only=2a798cc8409b4a1a9d26a8f652a3150b.
